# Involvement of ER stress in retinal cell death

**Published:** 2007-04-05

**Authors:** Masamitsu Shimazawa, Yuta Inokuchi, Yasushi Ito, Hiroshi Murata, Makoto Aihara, Masayuki Miura, Makoto Araie, Hideaki Hara

**Affiliations:** 1 Department of Biofunctional Molecules, Gifu Pharmaceutical University, Gifu, Japan; 2 Department of Ophthalmology, University of Tokyo School of Medicine, Tokyo, Japan; 3 Department of Genetics, Graduate School of Pharmaceutical Sciences, University of Tokyo, Tokyo, Japan

## Abstract

**Purpose:**

To clarify whether endoplasmic reticulum (ER) stress is involved in retinal cell death, using cultured retinal ganglion cells (RGC-5, a rat ganglion cell line transformed with E1A virus), and transgenic mice ER stress-activated indicator (ERAI) mice carrying a human XBP1 and venus a variant of green fluorescent protein (GFP) fusion gene.

**Methods:**

RGC-5 damage was induced by tunicamycin, and cell viability was measured by double nuclear staining (Hoechst 33342 and either YO-PRO-1 or propidium iodide). The expressions of glucose-regulated protein 78(GRP78)/BiP, the phosphorylated form of eukaryotic initiation factor 2α (p-eIF2α), and C/EBP-homologous (CHOP) protein after tunicamycin (in vitro or in vivo) or *N*-methyl-D-aspartate (NMDA; in vivo) treatment were measured using immunoblot or immunostaining. ERAI mice carrying the F-XBP1-DBD-venus expression gene were used to monitor ER-stress in vivo. Twenty-four hours after intravitreal injection of tunicamycin or NMDA, or after raising intraocular pressure (IOP), the retinal fluorescence intensity was visualized in anesthetized animals using an ophthalmoscope and in retinal flatmount or cross-section specimens using laser confocal microscopy.

**Results:**

Treatment with tunicamycin induced apoptotic cell death in RGC-5 and also induced production of ER stress-related proteins (BiP, the phosphorylated form of eIF2α, and CHOP protein). In vivo, tunicamycin induced retinal ganglion cell (RGC) loss and thinning of the inner plexiform layer, 7 days after intravitreal injection. In flatmounted retinas of ERAI mice, the fluorescence intensity arising from the XBP-1-venus fusion protein, indicating ER-stress activation, was increased at 24 h after tunicamycin, NMDA, or IOP elevation. In transverse cross-sections from ERAI mice, the fluorescence intensity was first increased in cells of the ganglion cell and inner plexiform layers at 12 and 24 h, respectively, after NMDA injection, and it was localized to ganglion and amacrine cells at 12 and 24 h, respectively, and to microglial cells at 72 h. BiP and CHOP were increased at 12 h after NMDA injection, and the increases persisted for the remainder of the 72 h observation period.

**Conclusions:**

These data indicate that ER-stress may play a pivotal role in RGC death, whether induced by NMDA or IOP elevation.

## Introduction

Endoplasmic reticulum (ER) stress is caused by a number of biochemical and physiological stimuli that result in the accumulation of unfolded proteins in the ER lumen, and it is closely associated with the neuronal cell injury caused by vascular and neurodegenerative diseases such as stroke, Alzheimer disease, and Parkinson disease [1-3]. However, little is known about the role, if any, of ER stress in retinal damage.

Retinal ganglion cell (RGC) death is a common feature of many ophthalmic disorders such as glaucoma, optic neuropathies, and retinovascular diseases, such as diabetic retinopathy and retinal vein occlusions. RGC death has been reported to occur via a variety of mechanisms involving, for example, oxidative stress [4], excitatory amino acids [5], nitric oxide (NO) [6], and apoptosis [7]. Glutamate, one of the excitatory amino acids, is the main neurotransmitter in the retinal signaling pathway. Excessive glutamate increases both intracellular Ca^2+^ and NO production through activation of the *N*-methyl-D-aspartate (NMDA)-type glutamate receptor, resulting in retinal cell death [8,9]. Recently, Uehara et al. [10] reported that in primary cortical culture, even mild exposure of NMDA induces apoptotic cell death. They demonstrated to be caused by an accumulation of polyubiquitinated proteins and increases in X box binding protein (XBP-1) mRNA splicing and C/EBP-homologous (CHOP) mRNA, representing activation of the unfolded-protein response (UPR) signaling pathway. They also found that protein-disulphide isomerase (PDI), which assists in the maturation and transport of unfolded secretory proteins, prevented the neurotoxicity associated with ER stress. They suggest that neurodegenerative disorders might be mediated by *S*-nitrosylation of PDI, which would reduce its enzymatic activity. Their results strongly suggest that activation of ER stress may participate in the retinal cell death occurring after NMDA receptor activation and/or ischemic insult. Hence, the purpose of the present study is to examine how ER stress might induce retinal damage both in vitro using cultured retinal ganglion cells (RGC-5, a rat ganglion cell line transformed using E1A virus) and in vivo (using ER stress-activated indicator (ERAI) transgenic mice, in which effective identification of cells under ER-stress conditions is possible in vivo, as described in our previous report) [11]. Use of ERAI mice should provide valuable information regarding the dynamics of ER stress-induced retinal damage.

## Methods

### Materials

Dulbeco's modified Eagles's medium (D-MEM) was purchased from Sigma-Aldrich (St. Louis, MO). The drugs used and their sources were as follows. Tunicamycin was obtained from Calbiochem (San Diego, CA) and Wako (Osaka, Japan). Isoflurane was acquired from Nissan Kagaku (Tokyo, Japan), and fetal bovine serum (FBS) was obtained from Valeant (Costa Mesa, CA).

### Retinal ganglion cell line (retinal ganglion cell-5) culture

Cultures of RGC-5 were maintained in D-MEM supplemented with 10% FBS, 100 U/ml penicillin (Meiji Seika Kaisha, Ltd., Tokyo, Japan), and 100 μg/ml streptomycin (Meiji Seika Kaisha, Ltd.) in a humidified atmosphere of 95% air and 5% CO_2_ at 37 °C. The RGC-5 cells were passaged by trypsinization every 3 days, as in a previous report [12].

### Cell viability assay after tunicamycin

RGC-5 cells were plated at a density of 1000 cells/well in 96-well culture plates (number 3072, Falcon®, Becton Dickinson and Company, Franklin Lakes, NJ). Twenty-four h later, cells were washed twice with D-MEM and then immersed in D-MEM supplemented with 1% FBS plus tunicamycin at 1 to 4 μg/ml. Twenty-four or forty-eight hours after the addition of tunicamycin, cell viability was measured using a single-cell digital imaging-based method employing fluorescent staining of nuclei. Briefly, cell death was assessed on the basis of combination staining with fluorescent dyes [namely, Hoechst 33342 (Molecular Probes, Eugene, OR) and either YO-PRO-1 (Molecular probes) or propidium iodide (PI; Molecular probes)]. Observations were made using an Olympus IX70 inverted epifluorescence microscope (Olympus, Tokyo, Japan). At the end of the above culture period, Hoechst 33342 and YO-PRO-1 or PI dyes were added to the culture medium at 8 μM, 0.1 μM, and 1.5 μM, respectively, for 30 min. Images were collected using a digital camera (Coolpix 4500, Nikon Corp., Tokyo, Japan). In a blind manner, a total of at least 400 cells per condition were counted using image-processing software (Image-J ver. 1.33f, National Institutes of Health, Bethesda, MD). Cell mortality was quantified by expressing the number of YO-PRO-1- or PI-positive cells as a percentage of the number of Hoechst 33342-positive cells.

### Animals

ER-stress-activated indicator (ERAI)-transgenic mice carrying the F-XBP1DDBD-venus expression gene [11] and their background wild-type mice (C57BL/6) aged 8-11 weeks or male adult ddY mice (Japan SLC, Hamamatsu, Japan) weighing 36-43 g for experiments other than the comparison with ERAI-transgenic mice were used, and were kept under controlled lighting conditions (12 h:12 h light/dark). All experiments were performed in accordance with the ARVO statement for the Use of Animals in Ophthalmic and Vision Research, and were approved and monitored by the Institutional Animal Care and Use Committee of Gifu Pharmaceutical University.

### Retinal damage induced by *N*-methyl-D-aspartate (NMDA)-, tunicamycin-, or intraocular pressure (IOP) elevation

Male mice were anesthetized with 3.0% isoflurane and maintained using 1.5% isoflurane in 70% N_2_O and 30% O_2_, delivered via an animal general anesthesia machine (Soft Lander, Sin-ei industry Co. Ltd., Saitama, Japan). The body temperature was maintained at 37.0 - 37.5 °C with the aid of a heating pad and heating lamp. Retinal damage was induced by injection (2 μl/eye) either of NMDA (Sigma-Aldrich) at 20 mM dissolved in 0.01 M phosphate-buffered saline (PBS) or of tunicamycin at 50 and 500 μg/ml, or (b) by acutely increasing the intraocular pressure (IOP). For NMDA- or tunicamycin-induced injury, the relevant agent was injected into the vitreous body of the left eye under the above anesthesia. In the IOP elevation model, the pupils were dilated with topical 2.5% phenylephrine hydrochloride and 1% tropicamide (Santen Pharmaceuticals Co. Ltd., Osaka, Japan). After topical instillation of 0.4% oxybuprocaine hydrochloride (Santen Pharmaceuticals Co. Ltd.), the anterior chamber was cannulated with a 32-gauge needle connected to a reservoir containing 0.9% NaCl. IOP was elevated by raising the height of the reservoir, maintaining a pressure of 100 mm Hg for 45 min. Retinal ischemia was confirmed by the blanching of the iris and retinal circulation. At the end of the elevated IOP period, the needle was removed, and reperfusion of the retinal vasculature was confirmed by ophthalmoscopic examination (KOM 300; Konan Inc., Nishinomiya, Japan). One drop of levofloxacin ophthalmic solution (Santen Pharmaceuticals Co. Ltd.) was applied topically to the treated eye after each procedure (intravitreal injection or ischemia-reperfusion).

### Monitoring endoplasmic reticulum (ER) stress using ERAI-transgenic mice

In anesthetized ERAI-transgenic or wild-type mice, retinal damage was induced by injection (2 μl/eye) of either NMDA at 20 mM or tunicamycin at 50 μg/ml into the vitreous body, or by elevating IOP to 100 mmHg for 45 min (see above). Twenty-four hours later, the fluorescence intensity arising from the XBP-1-venus fusion protein, which is translated from the F-XBP1DDBD-venus gene, was visualized in the retina of anesthetized animals using an ophthalmoscope (TRC-50; TOPCON, Tokyo, Japan) fitted with a fluorescence filter. In separate experiments, the distribution and time-course of changes in fluorescence intensity in the retina were measured in retinal flatmount and cross-section specimens using either laser confocal microscopy (Bio-Lad Laboratories, Inc, Hercules, CA) or epifluorescence microscopy (Power BX50; Olympus, Tokyo, Japan). At various times after the intravitreal injections (12, 24, and 72 h), eyes were enucleated, then fixed in 4% paraformaldehyde for 1 h or overnight at 4 °C as preparation for retinal flatmount and retinal cross-section, respectively. For the preparation of retinal flatmounts, detached retinas were flatmounted on slides (MAS COAT; MATSUNAMI GLASS IND., LTD., Osaka, Japan) by making radial incisions. They were then mounted under a coverslip and observed using the epifluorescence microscope. For the preparation of retinal cross-sections, fixed eyes were immersed in 20% sucrose for 48 h at 4 °C, and embedded in optimum cutting temperature (OCT) compound (Sakura Finetechnical Co., Ltd, Tokyo, Japan). Transverse, 10 μm thick cryostat sections were cut and placed onto slides (MAS COAT) under a coverslip, and observed using the laser confocal microscope.

### Immunoblotting

RGC-5 cells or mouse retinas were lysed using a cell-lysis buffer (RIPA buffer (R0278; Sigma) with protease (P8340; Sigma Aldrich) and phosphatase inhibitor cocktails (P2850 and P5726; Sigma), and 1 mM EDTA). Cell lysates were solubilized in SDS-sample buffer, separated on 10% SDS-polyacrylamide gels, and transferred to PVDF membrane (Immobilon-P; Millipore, Bedford, MA). Transfers were blocked for 1 h at room temperature with 5% Blocking One-P (Nakarai Tesque, Inc., Kyoto, Japan) in 10 mM Tris-buffered saline with 0.05% Tween 20 (TBS-T), then incubated overnight at 4 °C with the primary antibody. The transfers were then rinsed with TBS-T and incubated for 1 h at room temperature in horseradish peroxidase goat anti-rabbit or goat anti-mouse (Pierce, Rockford, IL) diluted 1:2000. The immunoblots were developed using chemiluminescence (Super Signal® West Femto Maximum Sensitivity Substrate; Pierce), and visualized with the aid of a digital imaging system (FAS-1000; Toyobo CO., LTD, Osaka, Japan). The primary antibodies used were as follows: mouse anti-BiP (BD Bioscience, San Jose, CA), rabbit anti-phospho-eIF2α (Ser51; Cell Signaling, Beverly, MA), rabbit anti-eIF2α (Cell Signaling), mouse anti-CHOP (Santa Cruz, Santa Cruz, CA), and rabbit anti-actin (Santa Cruz).

### Immunostaining

To clarify the distribution and localization of the XBP1-venus fusion protein in the retina of ERAI mice (as seen in the retinal flatmounts and cross sections), double-staining immunocytochemistry was performed. At various times after intravitreal injections (12, 24, and 72 h), eyes were enucleated, fixed in 4% paraformaldehyde overnight at 4 °C, immersed in 20% sucrose for 48 h at 4 °C, and embedded in optimum cutting temperature (OCT) compound (Sakura Finetechnical Co., Ltd, Tokyo, Japan). Transverse, 10 μm thick cryostat sections were cut and placed onto slides MAS COAT (MATSUNAMI GLASS IND., LTD.). Sections were subsequently processed for immunocytochemical localization using antibodies against CHOP (1:100 dilution in PBS; Santa Cruz), glucose-regulated protein 78(GRP78)/BiP (1:100 dilution in PBS), thymus cell antigen 1 (Thy-1; 1:100 dilution in PBS; Serotec Ltd, Oxford UK), microglia (OX-42, 1:100 dilution in PBS; Serotec Ltd), and amacrine cells (HPC-1/Syntaxin, 1:100 dilution in PBS; Santa Cruz). The sections were incubated either (a) with Alexa Fluor-568-conjugated secondary antibody (1:200 dilution in PBS; Molecular Probes, Eugene, OR) for 1 h at room temperature, mounted with a coverslip, and observed under a laser confocal microscope (Bio-Lad Laboratories, Inc), or (b) with biotin-conjugated secondary antibody for 1 h at room temperature, and visualized using a VECTOR M.O.M. Immunodetection kit (Vector, Burlingame, CA). Each image was taken using a digital camera (Coolpix 4500; Nikon, Tokyo, Japan) attached with epifluorescence microscope (Power BX50; Olympus).

### Histological analysis of mouse retina

Seven days after the NMDA or tunicamycin injection, eyeballs were enucleated for histological analysis. In mice under anesthesia, produced by an intraperitoneal injection of sodium pentobarbital (80 mg/kg), each eye was enucleated, then kept immersed for at least 24 h at 4 °C in a fixative solution containing 4% paraformaldehyde. Six paraffin-embedded sections (thickness, 3 μm) cut through the optic disc of each eye were prepared in a standard manner, and stained with hematoxylin and eosin. Retinal damage was evaluated as described previously, and three sections from each eye were used for the morphometric analysis. Light-microscope photographs were taken using a digital camera (Coolpix 4500, Nikon) and the cell counts in the ganglion cell layer (GCL) and the thickness of the inner plexiform layer (IPL) at a distance between 350 and 650 μm from the optic disc were measured on the images in a masked fashion by a single observer (Y.I.). Data from three sections (selected randomly from the six sections) were averaged for each eye, and the values obtained were used to evaluate the GCL cell count and the IPL thickness.

### Statistical analysis

Data are presented as the means±SEM. Statistical comparisons were made using a Student's *t*-test or Dunnett's test, by means of STAT VIEW version 5.0 (SAS Institute Inc., Cary, NC). P<0.05 was considered to be statistically significance.

## Results

### Retinal cell death and time-course of changes in endoplasmic reticulum (ER) stress-related protein induced by tunicamycin

**Figure 1:**
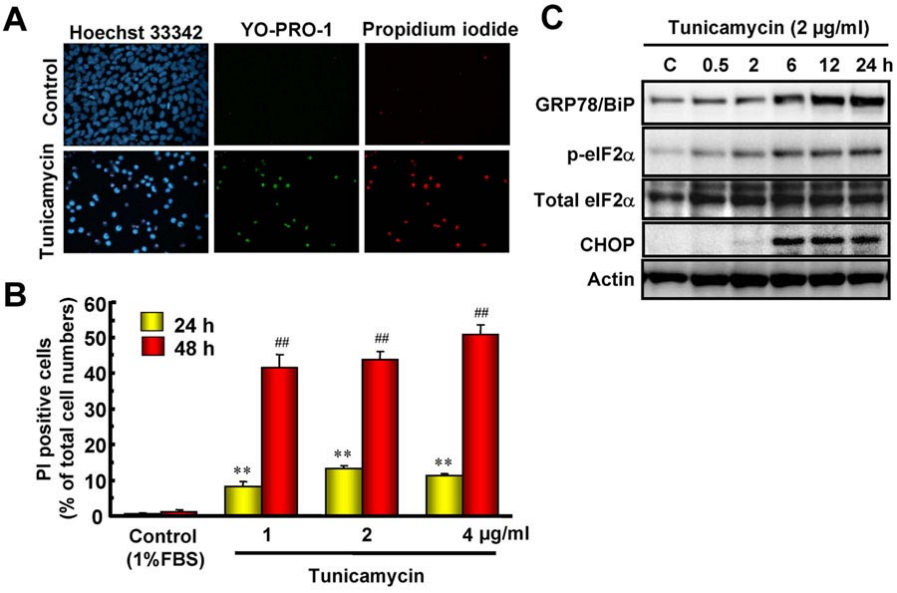
Retinal cell death and time-course of changes in endoplasmic reticulum (ER)-stress related proteins induced by tunicamycin. **A**: Representative fluorescence microscopy showing nuclear stainings for Hoechst 33342 (blue), YO-PRO-1 (green), and propidium iodide (PI, red) at 48 h after addition of tunicamycin at 1 μg/ml. **B**: The number of cells displaying PI fluorescence was counted at two time-points, and positive cells were expressed as the percentage of PI to Hoechst 33342. Each column represents the mean±SEM (n=6). Double asterisks and double hash marks; p<0.01 versus corresponding control group (Dunnett's test). **C**: Representative immunoblots showing the time-course of changes in protein levels (GRP78/BiP, phosphorylated-eIF2α, total eIF2α, and CHOP) after tunicamycin treatment at 2 μg/ml.

We examined whether tunicamycin treatment could induce cell death through ER stress in retinal ganglion cell using RGC-5. Representative fluorescence stainings of nuclei [using Hoechst 33342, YO-PRO-1, and propidium iodide (PI) dyes] are shown in Figure 1A. Vehicle-treated control cells displayed normal nuclear morphology and negative staining with both YO-PRO-1 dye (which stains early apoptotic and later-stage cells) and PI dye (which stains late-stage apoptotic cells; upper panels in Figure 1A). Treatment with tunicamycin led to shrinkage and condensation of nuclei, and to positive staining with each of these dyes (lower panels in Figure 1A). The number of cells exhibiting PI fluorescence was counted, and positive cells were expressed as the percentage of PI- to Hoechst 33342-positive cells (Figure 1B). After treatment with tunicamycin at 1, 2, or 4 μg/ml for 24 h, the percentages of PI-positive cells were 8.3±1.2% (n=6), 13.1±0.9% (n=6), and 11.3±0.6% (n=6), respectively, while in the non-treated control group the percentage was 0.5±0.2% (n=6). After treatment with tunicamycin at 1, 2, or 4 μg/ml for a longer time period (48 h), the corresponding values were 41.5±3.5% (n=6), 43.7±2.1% (n=6), and 50.7±2.6% (n=6), respectively (1.2±0.4% (n=6) for the non-treated control group). Time-course data for the changes in the protein levels of glucose-regulated protein (GRP)78/BiP, the phosphorylated form of eukaryotic initiation factor 2α (eIF2α), total eIF2α, and C/EBP-homologous protein (CHOP) occurring after tunicamycin treatment at 2 μg/ml are shown in Figure 1C. BiP, a biomarker of ER-stress, increased time-dependently throughout the 24 h tunicamycin treatment period, while actin levels remained unchanged. Treatment with tunicamycin time-dependently induced eIF2α phosphorylation, while total eIF2α levels were not changed during the 24 h observation period. CHOP was first detected at 6 h after addition of tunicamycin and persisted thereafter. These data indicate that treatment with tunicamycin can induce expressions of ER stress-related proteins and subsequent apoptotic cell death in RGC-5 culture in vitro.

### Intravitreal injection of tunicamycin induces retinal cell death in mice

**Figure 2:**
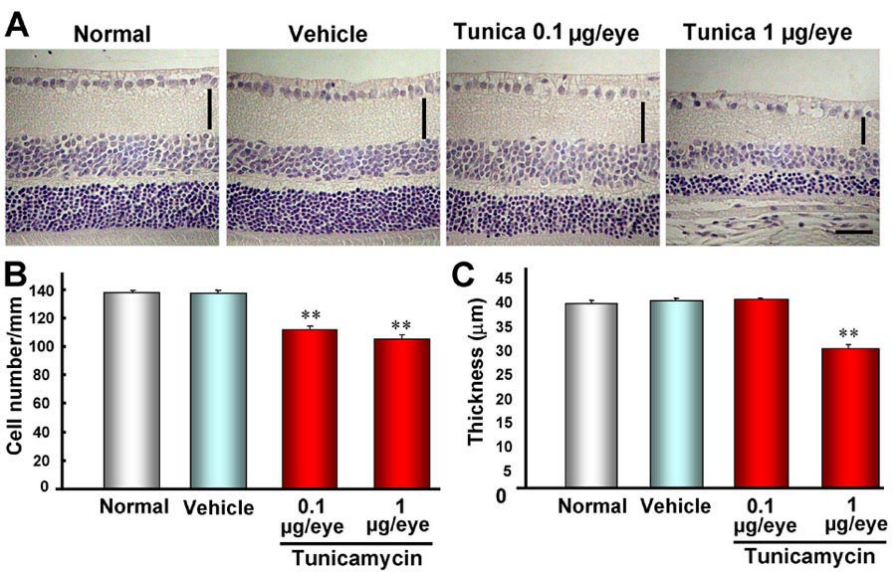
Intravitreal injection of tunicamycin induces retinal cell death in mice. **A**: Representative photographs showing non-treated normal retina, vehicle-treated retina, and low-dose (0.1 μg/eye) and high-dose (1 μg/eye) tunicamycin-treated retinas 7 days after intravitreal injection. Quantitative analysis of cell number in ganglion cell layer (**B**) and thickness of inner plexiform layer (IPL) **C**: Each column represents the mean±SEM (n=10). Double asterisks p<0.01 versus vehicle-treated control group (Dunnett's test). The horizontal scale bar represents 25 μm and the vertical bar indicates each thickness of IPL.

To clarify whether tunicamycin would induce retinal cell death in vivo, we examined the histological changes in the retina at 7 days after intravitreal injection of tunicamycin. As shown in Figure 2, intravitreal injection of tunicamycin at 0.1 μg/eye (a low dose) induced a significant loss of cells in the retinal ganglion cell layer (GCL), but no thinning of the inner plexiform layer (IPL; versus vehicle-treated retinas). At a high dose of 1μg/eye, tunicamycin significantly decreased both the cell count in GCL and the IPL thickness (versus the non-treated normal retina; Figure 2). On the other hand, no retinal damage was induced by intravitreal injection of an identical volume of vehicle (versus the non-treated retina). Together, these findings suggest that tunicamycin at 0.1 μg/eye (giving an estimated concentration in the vitreous body of approximately 10 μg/ml) induces retinal ganglion cell death at a concentration similar to that inducing exhibiting the apoptotic cell death in RGC-5 in vitro.

### Increase in XBP-1-venus fusion protein in the retina in ER stress-activated indicator (ERAI)-transgenic mice

**Figure 3:**
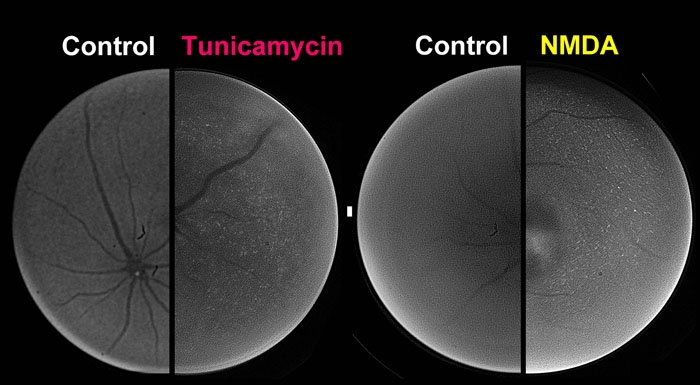
Non-invasive imaging of XBP-1-venus fusion protein in ERAI mouse retina in vivo. Twenty-four hours after intravitreal injection of either tunicamycin at 0.1 μg/eye or *N*-methyl-D-aspartate (NMDA) at 40 nmol/eye, the fluorescence intensity arising from XBP-1-venus fusion protein was visualized in the retinas of anesthetized animals using an ophthalmoscope fitted with a fluorescence filter.

**Figure 4:**
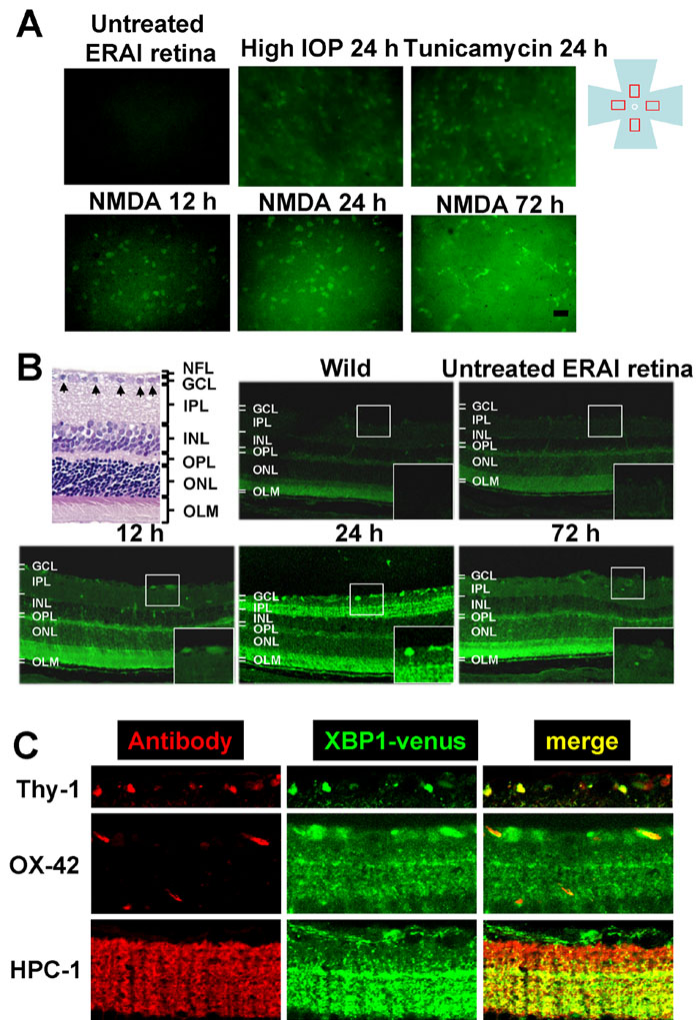
Expression and localization of XBP-1-venus fusion protein in ERAI mouse retinas after various types of retinal damage. **A**: Representative fluorescence photographs of increased XBP-1-venus fusion protein in ERAI mouse flatmounted retina after *N*-methyl-D-aspartate (NMDA), intraocular pressure (IOP) elevation, or tunicamycin insult. The fluorescence (green) arising from XBP-1-venus fusion protein was observed under an epifluorescence microscope. The scale bar represents 25 μm. **B**: Distribution of increased XBP-1-venus fusion protein in retinal cross-sections from ERAI mice after NMDA injection at 40 nmol/eye. The distribution of fluorescence (green) arising from XBP-1-venus fusion protein was observed under a laser confocal microscope. Each large box shows an enlargement of the area within the corresponding small box. **C**: Localization of XBP-1-venus fusion protein in ERAI mouse retina after NMDA injection. In the retinal nerve fiber layer (upper panels), Thy-1-positive cells (red) can be seen to merge with XBP-1-venus fusion protein (green). In the middle panels, OX-42 (a microglia marker)-positive cells (red) are partly merged with XBP-1-venus fusion protein (green). In the inner plexiform layer (lower panels), HPC-1 (an amacrine marker)-positive cells (red) are merged with XBP-1-venus fusion protein (green).

To investigate whether ER stress is induced in the mouse retina during the early stages of retinal damage in vivo, we used ERAI-transgenic mice carrying the F-XBP1DDBD-venus expression gene, which allows effective identification of cells under ER stress in vivo, as previously described by Iwawaki et al. [11]. Twenty-four h after intravitreal injection of tunicamycin at 0.1 μg or of *N*-methyl-D-aspartate (NMDA) at 40 nmol, the fluorescence intensity arising from the XBP-1-venus fusion protein was visualized in the retina of anesthetized animals (using an ophthalmoscope) as shown in Figure 3. Both tunicamycin and NMDA increased the fluorescence intensity of this protein, while little change in fluorescence intensity was observed in the control fellow eyes. For further elucidation of this phenomenon, the distribution and time-course of changes in the fluorescence intensity derived from the XBP-1-venus fusion protein were measured in retinal flatmount and transverse sections, as shown in Figure 4A,C. In the flatmounts, such stimulations as NMDA, an intraocular pressure (IOP) elevation, and tunicamycin all induced increases in fluorescence intensity at the time-points indicated in Figure 4A. In the NMDA-treated retinas of ERAI mice, the background fluorescence intensity was time-dependently increased in the period from 12 to 72 h, but little change was observed in the NMDA-treated retinas of wild-type mice.

These changes in background could reflect increases in the lower part of the ganglion cell layer, such as the inner plexiform layer and neuroepithelial layer, of the retina. In transverse sections, increases in fluorescence intensity were first observed in cells of the GCL and inner plexiform layer at 12 and 24 h, respectively, after NMDA injection, and the increases peaked in GCL cells at 24 h (Figure 4B). The increase in fluorescence had diminished at 72 h after the NMDA injection, but morphologically distinct cells (such as microglia cells) had appeared in GCL. On the other hand, the retinas of wild-type and non-treated ERAI mice showed a low fluorescence intensity (below background), while a slight fluorescence intensity was observed in the neuroepithelial layer of the retina (Figure 4B). These cells merged with Thy-1-positive cells (ganglion cells) and some OX-42-positive cells (microglia) in GCL, and with HPC-1-positive cells (amacrine cells) in IPL (Figure 4C). Together, these results suggest that XBP-1 splicing, representing activation of the ER-stress signal pathway, may be induced in retinal ganglion and amacrine and microglia cells during the early stages of retinal cell damage.

### Increases in GRP78/BiP and CHOP in mouse retina after NMDA injection

**Figure 5:**
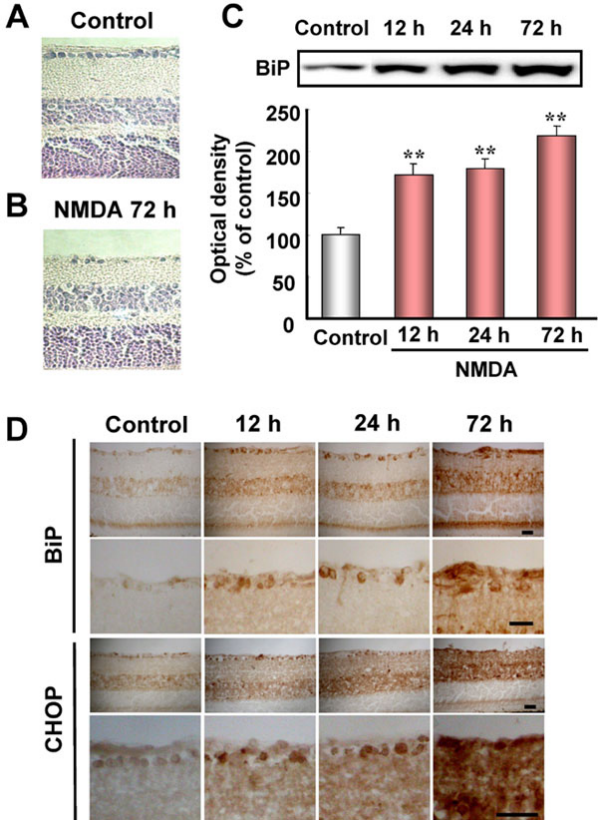
Increases in GRP78/BiP and CHOP in retinal extracts following stimulation by intravitreal injection of *N*-methyl-D-aspartate (NMDA) in mice. **A**, **B**: Representative photographs showing retinal cross-sections stained with hematoxylin and eosin after NMDA injection at 40 nmol/eye. **C**, upper panel: Representative immunoblots showing the time-course of changes in GRP78/BiP protein levels after intravitreal injection of NMDA. **C**, lower panel: Quantitative analysis of GRP78/BiP band densities. Data are expressed as mean±SEM (n=6) of values (in arbitrary units) obtained for single band density. Double asterisks represents p<0.01 versus vehicle-treated control group (Dunnett's test). **D**: Immunostainings for GRP78/BiP and CHOP in mouse retina after NMDA injection at 40 nmol. The scale bar represents 25 μm.

To clarify whether ER stress-related proteins other than XBP-1 are induced in the mouse retina by NMDA stimulation, we examined the changes in BiP, a biomarker of ER stress, in the retina after intravitreal injection of NMDA. As shown in Figure 5B, cell loss in GCL and thinning of IPL were observed at 72 h after NMDA injection (versus non-treated control retinas; Figure 5A). Using immunoblots, as shown in Figure 5C, we found that BiP was significantly increased at 12 h after the NMDA injection, and that the increase persisted for the remainder of the 72 h observation period. Next, we investigated the distribution and time-course of changes in GRP78/BiP and CHOP, a proapoptosis protein, after NMDA injection. In the non-treated control retina, slight immunoreactivities for BiP and CHOP were observed in a number of cells in GCL and IPL (Figure 5D). Increases in these immunoreactivities were observed in retinal ganglion cells at 12 h after NMDA injection, and time-dependent increases were noted in the inner retina (Figure 5D).

## Discussion

In the present study, we could detect pathological changes and time-dependent changes related to ER stress in retinal flatmount and transverse sections and in the retinas of living mice after retinal damage. Moreover, we demonstrated that ER stress signals were activated in the retina in vivo after tunicamycin, elevating IOP, or NMDA treatment.

Agents or conditions that adversely affect ER protein folding lead to an accumulation of unfolded or misfolded proteins in the ER, a condition defined as ER stress. ER stress can be induced by agents or conditions that interfere with (a) protein glycosylation (e.g., glucose starvation, tunicamycin, glucosamine), (b) disulfide-bond formation (e.g., DTT, homocysteine), (c) Ca^2+^ balance (A23187, thapsigargin, EGTA), and/or (d) a general overloading of the ER with proteins (e.g., viral or non-viral oncogenesis) [1,13,14]. However, little is known about any involvement of ER stress in retinal damage. In the present study, we found that tunicamycin induced the ER stress-associated proteins BiP, p-eIF2α, and CHOP in cultured RGC-5 cells. These protein levels started to increase at 2 to 6 h after the start of tunicamycin treatment, and increased time-dependently until 24 h after the start of the treatment, while apoptotic cell death with condensation and fragmentation of nuclei was observed 24 h later. BiP acts as an ER resident molecular chaperon that is induced by ER stress, and this protein refolds the unfolded proteins, thereby tending to maintain homeostasis in the ER [15,16]. Since CHOP is a member of the CCAAT/enhancer-binding protein family that is induced by ER stress and participates in ER-mediated apoptosis, CHOP may be a key molecule in retinal cell death [17]. In the present study, the phosphorylation of eIF2α was increased concomitantly with the increases in the expression of BiP and CHOP proteins, even through p-eIF2α might be expected to suppress protein synthesis. Boyce et al. [18] reported that selective inhibition of eIF2α dephosphorylation increases both p-eIF2α and CHOP protein. These data suggest that during ER stress, p-eIF2α (inactive form) is still able to stimulate the translation of ATF4 mRNA, thereby increasing the transcription of BiP or CHOP mRNA, but that enough unphosphorylated-eIF2α (active form) may remain to translate BiP and CHOP mRNAs to proteins. On the other hand, we found that staurosporine, which mediates mitochondrial dysfunctions resulting in apoptotic cell death, did not induce any increases in BiP and CHOP proteins in RGC-5 [unpublished data]. Taken together, these findings suggest that persistent ER stress may induce apoptotic cell death through the eIF2α-CHOP signal pathway in RGC-5.

Next, we tried to determine whether tunicamycin could induce retinal damage in vivo. Intravitreal injection of low-dose tunicamycin induced a significant loss of cells in the retinal ganglion cell layer (GCL), but no thinning of the inner plexiform layer (IPL). These findings suggest that retinal ganglion cells are more sensitive to ER stress-induced cell death than other retinal cells. High-dose tunicamycin significantly decreased both the cell count in GCL and the thickness of IPL. The concentration of tunicamycin in the vitreous body after an intravitreal injection of low-dose tunicamycin was estimated to be 10 μg/ml. The tunicamycin concentration achieved within the retina will have been less than this. Interestingly, in the present in vitro study, tunicamycin at 1 to 4 μg/ml induced cell death with an increase in ER-stress signals, suggesting that the in vivo concentration of tunicamycin in the retina was roughly similar to that employed in vitro. Use of tunicamycin at a high dose also led to decreases in IPL, INL (inner nuclear layer), and ONL (outer nuclear layer) in the retina. In guinea pigs, a single subcutaneous injection of tunicamycin at 0.4 mg/kg has been reported to induce hepatotoxicity with dilation of the cisternae of the ER [19]. Furthermore, Zinszner et al. [20] noted that in mice, a single sublethal intraperitoneal injection of tunicamycin (1 mg/kg) induces CHOP expression and subsequent severe histological damage with an increase in TUNEL-positive cells, and a characteristic transient renal insufficiency. They also found that CHOP-deficient mice show an attenuated increase in TdT-mediated dUTP nick-end labeling (TUNEL)-positive cells during the renal damage induced by tunicamycin. These findings suggest that in vivo, tunicamycin-induced retinal cell death is due, at least in part, to an ER-stress mechanism.

NMDA receptors may participate in the processes of excitotoxicity and neuronal death in the retina [21,22]. Previous studies have found that TUNEL-positive cells can be observed in the GCL and INL of the mouse retina at an early stage (within 24 h) after an intravitreal injection of NMDA [23,24]. The hallmark of NMDA-induced neuronal death is a sustained increase in the intracellular Ca^2+^ concentration accompanied by overactivation of vital Ca^2+^-dependent cellular enzymes [25]. Thus, the signal-transduction pathways for NMDA-mediated cell death in the retina are well studied, but not yet fully understood.

To illuminate the role and distribution of ER stress in vivo, we focused on the retina of ERAI mice. Information about the status of ER stress during the course of a given disease might be obtained by crossing an ERAI transgenic mouse (the indicator mouse for ER stress in living cells) with a mouse model of the human disease of interest. In flatmounted retinas, fluorescence was detected following various stimulations [tunicamycin, NMDA, and intraocular pressure (IOP) elevation]. To our knowledge, this is the first report demonstrating that NMDA and ischemic insult (elevating IOP), in addition to tunicamycin, can activate the ER stress signal (measured as the splicing of the XBP-1 and venus fusion gene in ERAI transgenic mice) in the retina in vivo. Interestingly, ER stress was also induced in the retina after a transient IOP elevation, defined as an ischemia-reperfusion model. It has been reported that this model exhibits retinal cell damage similar to that induced by NMDA, and that both of these examples of damage are protected against by MK-801, an NMDA receptor antagonist, and by NO synthetase-inhibitor treatment [8,26]. Although little is known about the precise mechanisms responsible for activation of ER stress after NMDA or IOP elevation (ischemia-reperfusion), both stimuli cause intracellular Ca^2+^ overload and increased NO production, resulting in apoptotic cell death. Several lines of study suggest that intracellular Ca^2+^ overload and excessive production of NO deplete Ca^2+^ in the ER, thereby resulting in ER stress [27,28]. Recently, Uehara et al. [10] reported that NO induces S-nitrosylation of protein-disulphide isomerase (PDI), an enzyme that assists in the maturation and transport of unfolded secretory proteins and thereby helps to prevent the neurotoxicity associated with ER stress. S-nitrosylated-PDI exhibits reduced enzymatic activity and induces cell death through the ER stress pathway. These mechanisms may contribute to the activation of ER stress in the retina after NMDA stimulation or IOP elevation. Accordingly, our findings may provide important new insights into the mechanisms underlying the retinal cell damage induced by NMDA and by ischemia-reperfusion. In transverse retinal sections, we observed an increase in fluorescence intensity within the cells of the ganglion cell layer (GCL) and inner plexiform layer (IPL) at 12 and 24 h, respectively, after NMDA injection. The cells displaying increased fluorescence were ganglion cells (at 12 h after the injection), amacrine cells in IPL (at 24 h), and microglia in GCL (at 72 h). These data indicate that ganglion cells may be more sensitive to ER stress than the other retinal cells examined.

To further clarify the participation of ER stress, we examined the changes in BiP and CHOP in the retina after NMDA-induced injury. We found (a) that NMDA induced BiP proteins in the retina at 12 h after its injection (on the basis of immunoblots), and (b) that, NMDA induced both BiP and CHOP in the retina (especially within retinal ganglion cells and INL) at 12 h after its injection (on the basis of our immunostaining results). The expression of the CHOP gene reportedly increases in the rat retina after intravitreal injection of NMDA [29]. Furthermore, Awai et al. [30] found that treatment with MK-801, an NMDA receptor antagonist, inhibited the increases in CHOP mRNA and protein in the mouse retina that are observed after intravitreal injection of NMDA, and moreover that CHOP-deficient mice were resistant to NMDA-induced retinal damage. However, CHOP-deficient mice partially suppressed the NMDA-induced cell death, and therefore other pathways, such as mitochondrial dysfunction, may be engaged in the retinal cell death. Collectively, the above results indicate that NMDA can cause ER stress in the retina, and that the neurotoxicity induced by NMDA is due in part to a mechanism dependent on CHOP protein induction through excessive ER stress.

In conclusion, we have identified a close association between ER stress and retinal damage, and our results suggest that the ER stress-signal pathway might be a good target in the treatment of retinal diseases.
